# Separation of Seven Polyphenols from the Rhizome of *Smilax glabra* by Offline Two Dimension Recycling HSCCC with Extrusion Mode

**DOI:** 10.3390/molecules23020505

**Published:** 2018-02-24

**Authors:** Wei Guo, Hongjing Dong, Daijie Wang, Bin Yang, Xiao Wang, Luqi Huang

**Affiliations:** 1 School of Pharmaceutical Sciences, Shandong University of Traditional Chinese Medicine, 4655 Daxue Road, Jinan 250355, China; weiguo1982@126.com; 2 Institute of Chinese Materia Medica, China Academy of Chinese Medical Sciences, Beijing 100700, China; ybinmm@126.com; 3 State Key Laboratory Breeding Base of Dao-di Herbs, National Resource Center for Chinese Materia Medica, China Academy of Chinese Medical Sciences, 16 Nanxiaojie, Beijing 100700, China; 4 Shandong Academy of Chinese Medicine, 7 Yanzishanxi Street, Jinan 250014, China; 5 Shandong Key Laboratory of TCM Quality Control Technology, Shandong Analysis and Test Center, Qilu University of Technology (Shandong Academy of Sciences), 19 Keyuan Street, Jinan 250014, China; donghongjing_2006@163.com (H.D.); wangdaijie@126.com (D.W.)

**Keywords:** *Smilax glabra*, polyphenols, separation, high speed countercurrent chromatography (HSCCC), recycling coupled extrusion mode

## Abstract

An offline two-dimensional recycling high-speed countercurrent chromatography (2D R-HSCCC) strategy with extrusion mode was developed for isolating polyphenols from the rhizome of *Smilax glabra*. Firstly, the ethyl acetate extract was divided into two fractions, Fr.1 and Fr.2, by silica gel column chromatography. Then, HSCCC was applied to separate polyphenols from the two fractions using a solvent system consisting of petroleum ether–ethyl acetate–methanol–water (1:3:0.5:5, *v*/*v*). Fifty milligrams of Fr.1 was separated by conventional HSCCC, yielding 5-*O*-caffeoylshikimic acid (**1**, 15.8 mg) and taxifolin (**2**, 4.8 mg). Offline 2D R-HSCCC with extrusion mode was used to separate Fr.2, and astilbin (**4**, 37.3 mg), neoisoastilbin (**5**, 8.8 mg), engeletin (**7**, 7.9 mg), and a mixture of two polyphenols were obtained from 100 mg of Fr.2. The mixture of two polyphenols was further separated by pre-HPLC, yielding neoastilbin (**3**, 15.2 mg) and isoastilbin (**6**, 9.9 mg). The purities of these seven compounds were all over 96.0%. Their structures were identified by MS and NMR. The results demonstrated that the strategy based on offline 2D R-HSCCC with extrusion mode was a powerful tool to separate the main compounds from the rhizome of *Smilax glabra* and valued for the preparative separation compounds with broad *K*-values and similar structures.

## 1. Introduction

*Smilax glabra* Roxb., belonging to Smilacaceae, is mainly distributed in Southwestern China. The rhizome of *S. glabra* is known as Tufuling in China. As one of the most popular traditional Chinese medicines, it is used for relieving dampness, detoxifying, and easing joint movement [1,2]. Pharmacological studies indicate that it has anti-inflammatory, anti-tumor, and immunomodulatory activities and protective effects against hepatocyte damage [3,4,5]. Polyphenols are bioactive ingredients that have not only anti-inflammatory activities but also higher content expression in the rhizome of *S. glabra*. However, these polyphenols have similar structures, especially including four chiral isomers. Different configurations might have different or even opposite pharmacology activities. Thus, a large number of compounds with high purities are needed, which can be used to study the anti-inflammatory substance foundation for the rhizome of *S. glabra* and mechanism of these ingredients. Several studies in which these polyphenols are separated by column chromatography [6,7,8] have been reported, showing that this process takes a long time and produces low yields. With advantages such as high sample recovery, large loading capacity, low risk of sample denaturation, and irreversible adsorption [9], conventional high-speed countercurrent chromatography (HSCCC) has been applied for the separation of polyphenols from the rhizome of *S. glabra*. However, because the chemical structures of the polyphenols were similar, (four of them were isomers, and the partition coefficients (*K*-value) of the polyphenols had small differences), only two purified compounds (astilbin and isoastilbin) of these isomers were obtained [10,11]. Thus, effective methods for separation of polyphenols from the rhizome of *S. glabra* need to be developed.

In recent years, offline two-dimension recycling HSCCC (2D R-HSCCC) has been successfully applied to natural product separation [12,13]. Compared with the conventional pattern, R-HSCCC can increase the separation factor so that it can separate compounds with close *K*-values. Due to the limited separation capacity of 1D HSCCC, compounds of similar structures are always eluted in one peak. Two-dimensional HSCCC is introduced to separate the co-eluted compounds for the second time. An online strategy requires two HSCCC apparatuses and possibly other materials, but an offline strategy does no require that the effluent be introduced into the second dimension online. Therefore, offline 2D R-HSCCC will have promising applications in the purification of natural compounds from a complex matrix.

**Figure 1:**
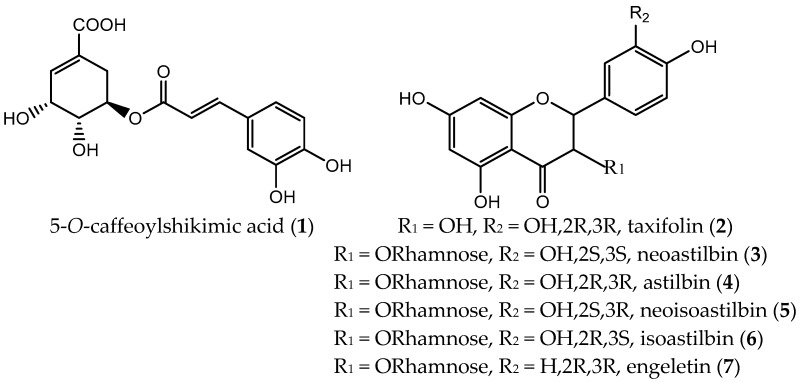
Structures of the compounds from the rhizome of *S. glabra*. (R: *Rectus*, right-handed; S: *Sinister*, left-handed).

In this work, offline 2D R-HSCCC was applied to separate the main bioactive polyphenols from the rhizome of *S. glabra*. Furthermore, due to the wide *K*-value range of the target compounds, extrusion mode was employed to rapidly separate compounds with high *K*-value. As a consequence, seven compounds were isolated with high purities and yields, and their structures are shown in Figure 1.

## 2. Results and Discussion

### 2.1. Optimization of Preliminary Separation and Two-Phase Solvent System

Being one of the most important steps in HSCCC separation, a suitable two-phase solvent system that can provide an ideal range of *K*-values for the targeted compounds needs to be selected. Generally, the most suitable range of *K*-values is expected to be between 0.5 and 2. A higher *K*-value might produce excessive broad peaks and an extended elution time; a lower *K*-value might lead to poor peak resolution. In addition, the separation factor between the adjacent components (α_1/2_ = *K*_1_/*K*_2_, *K*_1_ > *K*_2_) should be greater than 1.5 [9]. The solvent systems of chloroform–methanol–water, ethyl acetate–*n*-butyl alcohol–water, and petroleum ether–ethyl acetate–methanol–water (PEMW) were tested. The targets were biased towards the organic phase in the solvent systems of chloroform–methanol–water and ethyl acetate–*n*-butyl alcohol–water. Thus, the PEMW system was employed. Different volume ratios were tested, and the *K*-values are summarized in Table 1. 

**Figure 2:**
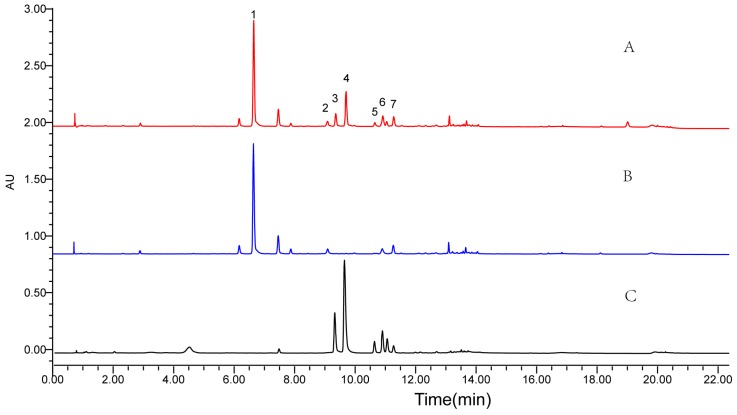
UPLC chromatograms of the EtOAc extract and the fractions (**A**: the EtOAc extract; **B**: Fr.1; **C**: Fr.2) Experimental conditions: an ACQUITY UPLC T3 column (100 mm × 2.1 mm i.d., 1.8 μm). Flow rate: 0.35 mL/min. Column temperature: 40 °C. Injection volume: 2 μL. Detection: 254 nm. UPLC conditions are as follows: acetonitrile (A) and water containing 0.1% (*v*/*v*) formic acid (B), the gradient elution mode was set as follows: 0–12 min, 5–23% A; 12–12.5 min, 23–31% A; 12.5–15.5 min, 31% A; 15.5–16 min, 31–43% A; 16–19 min, 43% A; 19–19.5 min, 43–100% A; 19.5–22.5 min, 100% A.

Results show smaller *K*-values of the targets presented in 1:3:1.5:5 and 1:3:1:5 (*v*/*v*) PEMW systems, while large *K*-values were obtained in 0.5:3.5:0.5:5 and 0.25:3:0.5:5 (*v*/*v*) systems. *K*-values of the 1:3:0.5:5 (*v*/*v*) mixture fell within a reasonable range. Therefore, a 1:3:0.5:5 (*v*/*v*) PEMW system could successfully elute the targets. However, α_1/5_ and α_2/4_ were much less than 1.5. In the HSCCC separation process, it was difficult to separate the seven peaks simultaneously. Thus, preliminary separation of **1** and **2** from **5** and **4** was inevitable. In view of this, the EtOAc extract was separated by silica gel column chromatography to produce Fr.1 and Fr.2. The chromatograms of the EtOAc extract and the fractions are shown in Figure 2. Peaks 1–7 correspond to 5-*O*-caffeoylshikimic acid (**1**), taxifolin (**2**), neoastilbin (**3**), astilbin (**4**), neoisoastilbin (**5**), isoastilbin (**6**), and engeletin (**7**), respectively.

### 2.2. Optimization of HSCCC Separation Process

**Figure 3:**
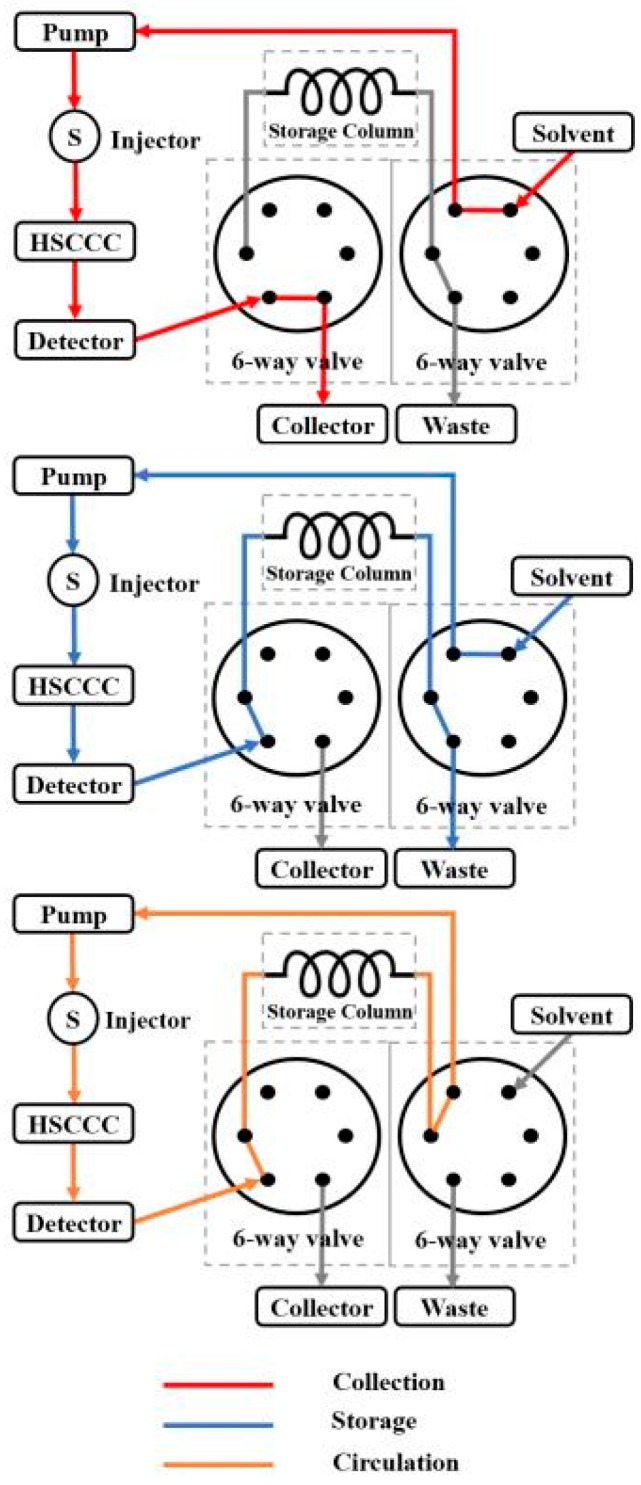
The switch sketch of three different patterns.

**Figure 4:**
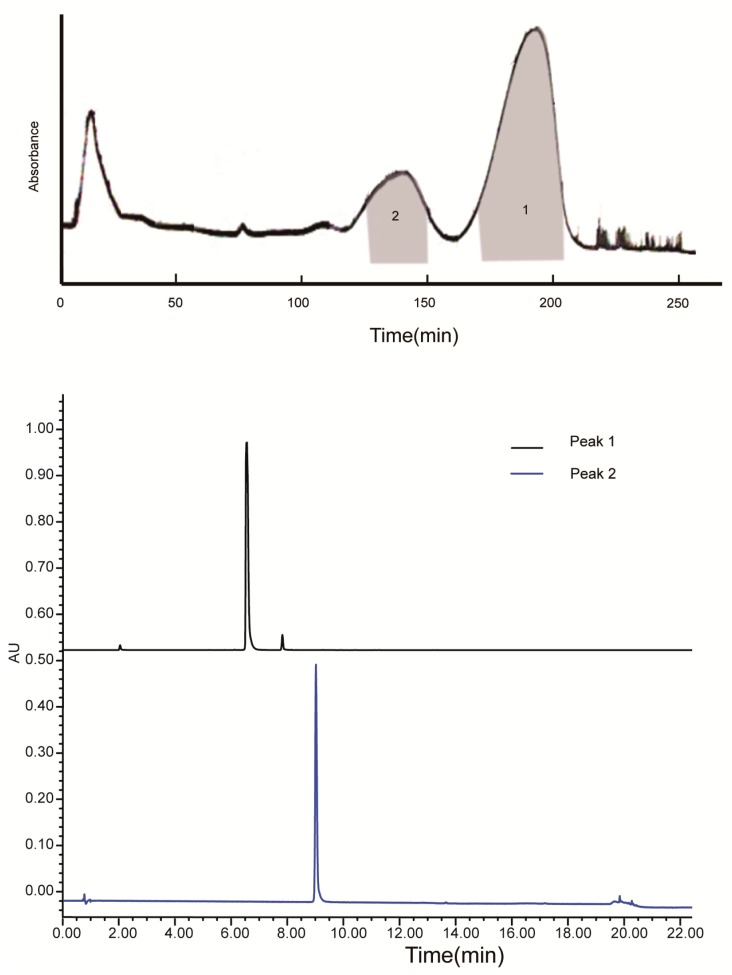
HSCCC chromatogram of Fr.1 and HPLC chromatogram of the two HSCCC fractions (Peak 1: 5-*O*-caffeoylshikimic acid; Peak 2: taxifolin). HSCCC condition: solvent system: PEMW (1:3:0.5:5, *v*/*v*); mobile phase: lower phase; stationary phase: upper phase; revolution speed: 850 rpm; sample loading: 50 mg of Fr.1 dissolved in 10 mL of lower phase; detection wavelength: 254 nm; flow rate: 2 mL/min; separation temperature: 25 °C; retention of the stationary phase: 62.1%.

Fr.1 was separated by conventional HSCCC (the connection sketch of conventional HSCCC is shown in the “Collection” pattern of Figure 3). The HSCCC chromatogram of Fr.1 and HPLC chromatogram of the two HSCCC fractions are presented in Figure 4, which reveal that 5-*O*-caffeoylshikimic acid (15.8 mg, 96.4%) and taxifolin (4.8 mg, 98.1%) were obtained from Fr.1.

**Figure 5:**
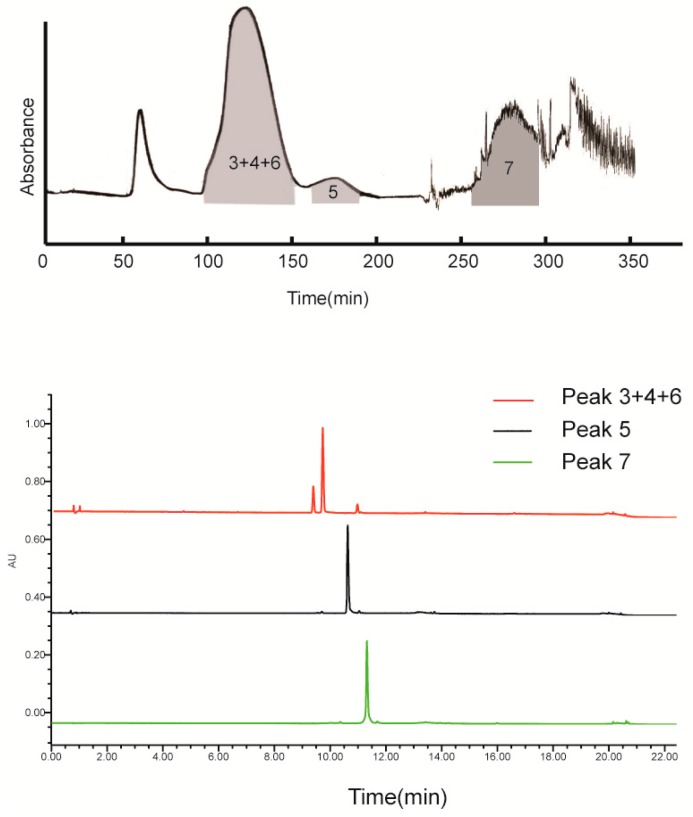
1D HSCCC chromatogram of Fr.2 and HPLC chromatogram of the three HSCCC fractions (Peak 3 + 4 + 6: the mixture of neoastilbin, astilbin, and isoastilbin; Peak 5: neoisoastilbin; Peak 7: engeletin). HSCCC condition was the same as that of Figure 4.

**Figure 6:**
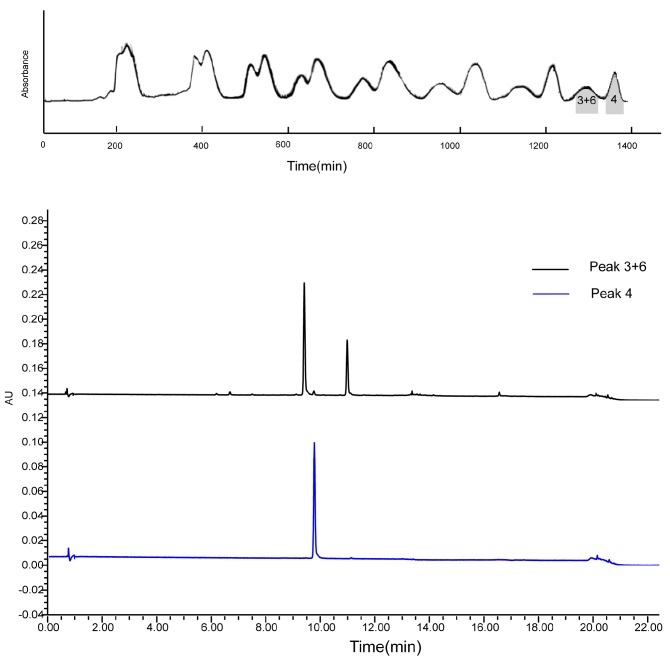
2D R-HSCCC chromatogram of Fr.2 and HPLC chromatogram of the two HSCCC fractions (Peak 3 + 6: the mixture of neoastilbin and isoastilbin; Peak 4: astilbin). HSCCC conditions were the same as it in Figure 4.

Fr.2 was separated by 2D R-HSCCC with extrusion mode. The 1D HSCCC chromatogram of Fr.2 is shown in Figure 5. The 2D HSCCC chromatogram of Fr.2 is shown in Figure 6. As can be seen in Table 1, the *K*-value of engeletin was too large. If a conventional HSCCC is used, the separation process will be time-consuming. Hence, an extrusion mode was used to rapidly separate engeletin. *K*-values of neoastilbin, astilbin, and isoastilbin were too close to be separated only by altering ratio of the solvent system. Thus, recycling elution mode was employed for the separation. In this HSCCC separation process, recycling proved suitable for compounds with close *K*-values. After the eighth cycle, the mixture of neoastilbin and isoastilbin was completely separated from astilbin. In addition, an offline 2D process was executed to prevent the residual compounds in the stationary phase from overlapping with the targets during the recycling process. Due to the very close *K*-values of neoastilbin and isoastilbin, extending the cycle time would otherwise be unsuccessful. Thus, neoastilbin and isoastilbin were prepared by prep-HPLC. Finally, five compounds were obtained from Fr.2: neoastilbin (15.2 mg, 98.2%), astilbin (37.3 mg. 98.6%), neoisoastilbin (8.8 mg.97.1%), isoastilbin (9.9 mg, 98.9%), and engeletin (7.9 mg, 98.3%). Compared with previous reports [6], the yield of all seven compounds increased by five to eight times. 

## 3. Materials and Methods 

### 3.1. Apparatus and Materials 

HSCCC separation was conducted on a TBE-300C (Tauto Biotechnique, Shanghai, China). The apparatus consists of a 300 mL PTFE multilayer coil (diameter of the PTFE tube was 1.9 mm) and a 20 mL manual sample loop. The rotation speed could be adjustable from 0 to 1000 rpm. The system was also equipped with a TBP-5002 constant-flow pump (Tauto Biotechnique, Shanghai, China), an 8823A-UV Monitor at 254 nm (Beijing Emilion Technology, Beijing, China), a Model 3057 portable recorder (Yokogawa, Sichuan Instrument Factory, Sichuan, China), and a DC-0506 low constant temperature bath (Tauto Biotechnique, Shanghai, China) to maintain a temperature of 25 °C. The outlet of the detector and the inlet of the pump were connected by two 6-way valves (see Figure 3). The “Collection” pattern shows the connection of conventional HSCCC. Offline 2D R-HSCCC was achieved with three patterns: “Collection”, “Storage”, and “Circulation”. The three patterns were enforced by turning the two 6-way valves. The “Collection” pattern was used to collect the targeted compound. By switching the two 6-way valves as per the “Storage” pattern, the storage tube was connected to an HSCCC separation system. The “Storage” pattern was used to store samples temporarily in a storage tube in preparation for further separation. The “Circulation” pattern was used to connect the inlet of the pump and the outlet of the detector to form a closed loop when recycling of the mobile phase began. 

A Waters Acquity UPLC^TM^ DAD system (Waters Corporation, Milford, CA, USA) was employed for the sample analysis. The column used was an ACQUITY UPLC T3 column (100 mm × 2.1 mm, i.d., 1.8 μm, Waters, MA, USA) coupled with a VanGuardTM T3 pre-column (10 mm × 2.1 mm, i.d.,1.8 μm, Waters, MA, USA). The prep-HPLC system consisted of a SHIMADZU LC-6AD prep-LC controller equipped with an SPD-M20AVP UV–Vis photodiode array detection (DAD) system and a Class-VP-LC work station (Shimadzu, Kyoto, Japan). NMR spectra were performed on a Bruker AV-400 spectrometer (Bruker BioSpin, Rheinstetten, Germany). ESI-MS experiments were performed on an Agilent 6520 Q-TOF (Agilent, Santa Clara, CA, USA).

All organic solvents used for sample preparation and HSCCC were of analytical grade (Sinopharm Chemical Reagent Co., Ltd., Shanghai, China). Methanol and acetonitrile used for HPLC analysis and prep-LC were purchased from Fisher Scientific (Fair Lawn, NJ, USA). Silica gel was purchased from Qingdao Haiyang chemical Co., Ltd. (Qingdao, China).

*S. glabra* was collected in August 2017 from the city of Guiyang, Guizhou province, China, and identified by Prof. Xiaohua Jin (Institute of Botany, the Chinese Academy of Sciences) and Prof. Yan Jin (National Resource Center for Chinese Materia Medica, China Academy of Chinese Medical Sciences). A voucher specimen was deposited in the Herbarium of Pharmacognosy, the National Resource Center for Chinese Materia Medica, China Academy of Chinese Medical Sciences.

### 3.2. Preparation of the Sample

The powdered rhizome of *S. glabra* (1 kg) was extracted by ultrasonic treatment with methanol (10 L, twice) for 1 h. After the extracts were evaporated, the residue (160 g) was suspended in 300 mL of water and then successively partitioned with dichloromethane (300 mL × 3) and ethyl acetate (EtOAc, 300 mL × 3). The EtOAc-soluble portion (15 g), after it was evaporated, was subjected to silica gel (200–300 mesh, 150 g) column chromatography and eluted with 95:5:0.5 *v*/*v* and 90:10:0.5 *v*/*v* mixtures of chloroform–methanol–formic acid to produce Fr.1 (101 mg) and Fr.2 (253 mg), respectively. Fr.1 and Fr.2 were evaporated for subsequent HSCCC separation. 

### 3.3. Selection of the Two-Phase Solvent System

The solvent system for HSCCC separation was selected by comparing the difference of partition coefficients (*K*) of the target compounds in various solvent systems. The test solvent system was equilibrated in a separatory funnel at room temperature, and the two phases were separated shortly before use. The sample solution was prepared by dissolving the EtOAc extract (0.05 g) in the solvent mixture of lower phase and upper phase (1:1, *v*/*v*). The *K*-value was expressed as the peak area of the compound in the upper phase divided by that in the lower phase. 

### 3.4. Preparation of the Two-Phase Solvent System and the Sample Solution

After the solvent system was thoroughly equilibrated in a separatory funnel and left overnight at room temperature, the system was divided into two phases. The two phases were then degassed via supersonic waves for 30 min before use. The upper organic phase was used as the stationary phase, and the lower aqueous phase was used as the mobile one. The sample solution of Fr.1 was prepared by dissolving 50 mg in 10 mL of lower phase and upper phase (1:1, *v*/*v*). Fr.2 was prepared by dissolving 100 mg in 10 mL of lower phase and upper phase (1:1, *v*/*v*).

### 3.5. The HSCCC Separation Procedure

Fr.1 was separated only by conventional HSCCC. The procedure was executed as follows: the separation column was filled with the upper phase at 20 mL/min, and the apparatus was then rotated at 850 rpm. The lower phase was pumped into the column at a flow rate of 2.0 mL/min. After equilibrium was established, the sample solution was injected into the sample loop. Fractions were manually collected by the HSCCC chromatogram. The retention of the stationary phase was defined as the stationary phase relative to the total column capacity after separation.

Fr.2 was separated by 2D R-HSCCC with extrusion mode. Firstly, the 6-way valves were adjusted to the “Collection” pattern (Figure 3) until the mixture of neoastilbin, astilbin, and isoastilbin was detected; at this point, the valves were switched to the “Storage” pattern so that the mixture of neoastilbin, astilbin, and isoastilbin was stored in a storage column for subsequent recycling separation. When neoisoastilbin was detected, the “Collection” pattern was assumed so that the compound could be collected. After neoisoastilbin was collected, rotation of the column ceased. The extrusion mode was used here to extrude the rest of the stationary phase by pumping the upper phase into the column at a flow rate of 10 mL/min engeletin was manually collected according to the elution profile. After engeletin was collected, the apparatus was rotated at 850 rpm. The lower phase was then pumped into the column at a flow rate of 2.0 mL/min. After equilibrium was established, the “Circulation” pattern was assumed. The mixture of neoastilbin, astilbin, and isoastilbin was then released into the recycling tube. When the targets were sufficiently separated through eight cycles, the mixture of neoastilbin, isoastilbin, and astilbin were collected by assuming the “Collection” pattern. The mixture of neoastilbin and isoastilbin was prepared for subsequent prep-HPLC separation. 

### 3.6. Prep-HPLC Separation 

Prep-HPLC separation was performed with YEG-C18 column (250 × 10 mm i.d., 5 μm) with a solvent of methanol–water (30:70) at a flow rate of 5 mL/min and monitored at 254 nm.

### 3.7. Analysis of Separated Compounds

The crude fraction and peak fraction were analyzed via UPLC. The mobile phase was acetonitrile (A) and water containing 0.1% formic acid (B); the gradient elution mode was set as follows: 0–12 min, 5–23% A; 12–12.5 min, 23–31% A; 12.5–15.5 min, 31% A; 15.5–16 min, 31–43% A; 16–19 min, 43% A; 19–19.5 min, 43–100% A; 19.5–22.5 min, 100% A. Flow rate was 0.35 mL/min.

### 3.8. Identification of Separated Compounds

Identification of separated compounds was carried out by ESI-MS, ^1^H-NMR, and ^13^C-NMR. The data was analyzed in comparison with the literature [6,7,8,14]. The data for each compound is as follows.

*5-O-Caffeoylshikimic acid* (Compound **1**): Pale brown powder; ESI-MS (*m*/*z*): 335.1 [M − H]^−^, 336.1 [M + H]^+^. ^1^H-NMR (400 MHz, DMSO-*d*_6_) δ(ppm): 7.47 (1H, d, *J* = 15.6 Hz, H-9), 7.05 (1H, d, *J* = 1.6 Hz, H-2′), 7.01 (1H, dd, *J* = 8, 1.6 Hz, H-6′), 6.76 (1H, d, *J* = 8 Hz, H-5′), 6.68 (1H, m, H-2), 6.26 (1H, d, *J* = 16 Hz, H-8), 5.10 (1H, dt, H-5), 4.30 (1H, br. t, H-3), 3.77 (1H, dd, *J* = 7.2, 4.0 Hz, H-4), 2.66 (1H, dd, *J* = 18.4, 4.8 Hz, H-6b), 2.20 (1H, dd, *J* = 18.4, 4.8 Hz, H-6a); ^13^C-NMR (100 MHz, DMSO-*d*_6_) 168.1 (COOH), 166.6 (C-7), 149.0 (C-4′), 146.0 (C-9), 145.7.0 (C-3′),139.0 (C-2), 128.5 (C-1), 126.0 (C-1′), 121.9 (C-6′), 116.2 (C-5′), 115.4 (C-2′), 114.5 (C-8), 70.4 (C-5), 68.2 (C-4), 65.9 (C-3), 28.2 (C-6).

*Taxifolin* (Compound **2**): White powder; ESI-MS (*m*/*z*): 303.1 [M − H]^−^, 305.1 [M + H]^+^. ^1^H-NMR (400 MHz, DMSO-*d*_6_) δ(ppm): 6.95 (1H, d, *J* = 1.8 Hz, H-2′ ), 6.84 (1H, dd, *J* = 7.8, 1.8 Hz, H-6′), 6.79 (1H, d, *J* = 7.8 Hz, H-5′), 5.88 (2H, s, H-6, 8), 4.91 (1H, d, *J* = 11.4Hz, H-2), 4.49 (1H, d, *J* = 11.4 Hz, H-3); ^13^C-NMR (100 MHz, DMSO-*d*_6_) δ: 197.0 (C-4), 167.4 (C-7), 163.9 (C-5), 163.1 (C-9), 145.8 (C-4′), 144.9 (C-3′), 128.5 (C-1′),120.9 (C-6′), 119.5 (C-2′), 114.6 (C-5′), 100.5 (C-10), 96.0 (C-6), 94.9 (C-8), 83.7 (C-2), 72.7 (C-3).

*Neoastilbin* (Compound **3**): White powder; ESI-MS (*m*/*z*): 449.1 [M − H]^−^, 451.1 [M + H]^+^. ^1^H-NMR (400 MHz, DMSO-*d*_6_) δ(ppm): 6.90 (1H, s, H-2′), 6.71 (2H, s, H-5′, 6′), 5.90 (1H, d, *J* = 2 Hz, H-6), 5.85 (1H, d, *J* = 2 Hz, H-8), 5.10 (1H, d, *J* = 11.2Hz, H-2), 4.73 (1H, d, *J* = 11.2 Hz, H-3), 4.94 (1H, s, H-1″), 3.77 (1H, s, H-2″), 3.15 (1H, dd, *J* = 9.2, 2.8 Hz, H-3″), 3.03 (1H, t, H-4″), 2.26 (1H, m, H-5″), 0.80 (3H, d, *J* = 6.0 Hz, H-6″); ^13^C-NMR (100 MHz, DMSO-*d*_6_) δ: 196.7 (C-4), 167.9 (C-7),163.9 (C-5), 162.9 (C-9), 146.5 (C-4′), 145.7 (C-3′), 128.0 (C-1′), 119.9 (C-6′), 115.4 (C-5′), 101.7 (C-10), 96.7 (C-6), 95.6 (C-8), 75.3 (C-3), 71.8 (C-4″), 70.7 (C-3″), 70.6 (C-2″), 69.4 (C-5″), 18.1 (C-6″).

*Astilbin* (Compound **4**): White powder; ESI-MS (*m*/*z*): 449.1 [M − H]^−^, 451.1 [M + H]^+^. ^1^H-NMR (400 MHz, DMSO-*d*_6_) δ(ppm): 6.90 (1H, s, H-2′), 6.74 (2H, s, H-5′, 6′), 5.89 (2H, dd, *J* = 8.8, 1.6 Hz, H-6, 8), 5.25 (1H, d, *J* = 9.6 Hz, H-2), 4.65 (1H, dd, *J* = 9.6 Hz, H-3), 4.54 (1H, s, H-1″), 4.03 (1H, br. s, H-2″), 3.89 (1H, dq, *J* = 9.2, 1.2 Hz, H-5″), 3.13 (1H, t, *J =* 8 Hz, H-4″), 3.05 (1H, dd, *J =* 9.2, 3.2 Hz, H-3″), 1.05 (3H, d, *J* = 6.0 Hz, H-6″); ^13^C-NMR (100 MHz, DMSO-*d*_6_) δ: 194.9 (C-4), 167.6 (C-7),163.9 (C-5), 162.6 (C-9), 146.4 (C-4′), 145.6 (C-3′), 127.4 (C-1′), 119.4 (C-6′), 115.8 (C-5′), 100.5 (C-10), 96.5 (C-6), 95.6 (C-8), 76.1 (C-3), 72. (1C-4″), 70.9(C-3″), 70.6 (C-2″), 69.5(C-5″), 18.2 (C-6″).

*Neoisoastilbin* (Compound **5**): White powder; ESI-MS (*m*/*z*): 449.1 [M − H]^−^, 451.1 [M + H]^+^. ^1^H-NMR (400 MHz, DMSO-*d*_6_) δ(ppm): 6.90 (1H, d, *J =* 1.2 Hz, H-2′), 6.74 (2H, dd, *J* = 6.0, 2.0 Hz, H-6′, 5′), 5.92 (2H, d, *J* = 7.2 Hz, H-6, 8), 5.46 (1H, d, *J* = 1.6 Hz, H-2), 4.5 (1H, d, *J* = 1.6 Hz, H-3), 4.10 (1H, d, *J* = 2.0 Hz, H-1″), 3.59 (1H, s, H-2″), 3.45 (1H, m, H-5″), 3.25 (1H, s, H-3″), 3.10 (1H, m, H-4″), 1.01 (3H, d, *J* = 6 Hz, H-6″); ^13^C-NMR (100MHz, DMSO-*d*_6_) δ: 192.9 (C-4), 168.0 (C-7), 164.4 (C-5), 163.0 (C-9), 145.8 (C-4′), 145.5 (C-3′), 127.0 (C-1′), 118.3 (C-6′), 115.7 (C-5′), 114.9 (C-2′),101.1 (C-1″), 100.9 (C-10), 96.6 (C-6), 95.5 (C-8), 80.5 (C-2), 75.9(C-3), 72.1 (C-4″), 70.9 (C-3″),70.4 (C-2″), 69.5 (C-5″), 17.9 (C-6″).

*Isoastilbin* (Compound **6**): White powder; ESI-MS (*m*/*z*): 449.1 [M − H]^−^, 451.1 [M + H]^+^. ^1^H-NMR (400 MHz, CD_3_OD) δ(ppm): 6.94 (1H, s, H-2′), 6.83 (1H, d, *J =* 2.4, H-6′), 6.8 (1H, d, *J =* 2.4 Hz, H-5′), 5.97 (1H, s, H-6), 5.92 (1H, s, H-8), 5.42 (1H, s, H-2), 4.96 (1H, s, H-3), 4.18 (1H, s, H-1″), 3.67 (1H, s, H-2″), 3.45 (1H, dd, *J* = 9.6, 3 Hz, H-3″), 3.35 (1H, dd, *J =* 9.6 Hz, H-5″), 3.03 (1H, t, *J =* 9.6, H-4″), 0.93 (3H, d, *J =* 6 Hz, H-6″); ^13^C-NMR (100 MHz, CD_3_OD) δ: 194.6 (C-4), 168.7 (C-7), 164.8 (C-5), 163.1 (C-9), 145.3 (C-4′), 145.0 (C-3′), 127.3 (C-1′), 118.0(C-6′), 115.0 (C-5′), 113.8 (C-2′), 100.4 (C-1″), 98.8 (C-10), 96.0 (C-6), 96.8 (C-8), 80.7 (C-2), 74.2 (C-3), 71.9 (C-4″), 70.6 (C-3″), 70.5 (C-2″), 69.1 (C-5″), 16.4 (C-6″).

*Engeletin* (Compound **7**): White powder; ESI-MS (*m*/*z*): 433.1 [M − H]^−^, 435.1 [M + H]^+^. ^1^H-NMR (400 MHz, DMSO-*d*_6_) δ(ppm): 7.35(2H, d, *J* = 8.4 Hz, H-2′, 6′), 6.77 (2H, d, *J* = 8.4 Hz, H-3′, 5′), 5.77 (2H, d, *J* = 8 Hz, H-8, 6), 5.23 (1H, d, *J* = 9.6 Hz, H-2), 4.67 (1H, d, *J* = 14.4 Hz, H-3), 3.95 (1H, s, H-1″), 3.15 (1H, m, H-4″), 3.05 (1H, m, H-5″), 1.17 (3H, d, *J* = 6.5 Hz, H-6″); ^13^C-NMR (100 MHz, DMSO-*d*_6_) δ: 164.0 (C-5), 162.5 (C-9), 158.3 (C-4′), 129.5 (C-2′, 6′), 128.5 (C-1′), 115.6 (C-3′, 5′), 100.7 (C-1″), 102.2 (C-10), 97.4 (C-8), 96.1 (C-6), 76.4 (C-2), 72.3 (C-3), 71.0 (C-4″), 70.6 (C-3″), 69.5 (C-5″), 18.2(C-6″).

## 4. Conclusions

In this experiment, offline 2D recycling HSCCC with extrusion mode was easily established for the separation of main bioactive components from the rhizome of *S. glabra*. Seven polyphenols, namely, 5-*O*-caffeoylshikimic acid, taxifolin, neoastilbin, astilbin, neoisoastilbin, isoastilbin, and engeletin, were successfully isolated with high purities (over 96%). Considering the similar structures of these compounds (including four chiral isomers), conventional HSCCC, restricted by relatively limited peak capacity, could not achieve satisfactory separation. An offline 2D recycling HSCCC strategy with extrusion mode improved column capacity, peak resolution, and the utilization rate of the solvent. Although pre-purification was a prerequisite in our experiment, the strategy proved to effectively separate targets with broad *K*-values and similar structures. It is believed that offline 2D recycling will serve as an efficient pattern for the separation of natural products.

## Figures and Tables

**Table 1:** *K*-values of target compounds in different two-phase solvent systems.

Solvent System (PEMW, *v*/*v*)	*K*-Value						
	1	2	3	4	5	6	7
1:3:1.5:5	0.40	0.32	0.23	0.29	0.33	0.24	0.79
1:3:1:5	0.64	0.59	0.36	0.42	0.46	0.41	1.31
1:3:0.5:5	1.67	1.10	0.89	1.11	1.67	0.90	4.91
0.5:3.5:0.5:5	2.21	2.04	1.44	1.81	2.19	1.58	5.44
0.25:3:0.5:5	2.55	2.16	1.66	2.00	2.32	1.71	6.16
